# Formulation and characterization of reduced fat muffins using a plant based fat replacer

**DOI:** 10.1007/s13197-024-06045-6

**Published:** 2024-09-28

**Authors:** Mehak Ahsan, Abeera Moin, Humaira Ashraf, Alvina Khan, Angelo Maria Giuffrè

**Affiliations:** 1 Department of Food Science and Technology, Jinnah University for Women, Nazimabad, Karachi, 74600 Pakistan; 2 Department of Food Science and Technology, University of Karachi, Karachi, 75270 Sindh Pakistan; 3 Department of AGRARIA, University of Studies “Mediterranea” of Reggio Calabria, Via Dell’Università, 25, 89124 Reggio Calabria, Italy

**Keywords:** Sago, Muffins, Flour, Microstructure, Texture

## Abstract

Obesity and associated health alarms have encouraged increased awareness in developing healthier food alternatives, such as low-fat bakery products. This study explores a sustainable plant-based approach to formulate low-fat muffins by partially replacing butter with sago flour at levels of 25, 37, and 50% (w/w). The research designed to assess the physicochemical and pasting properties of wheat-sago flour composites and their impact on the texture, color, post-baking attributes, and sensory characteristics of the muffins. Results presented that the ash content of the fat-reduced muffins significantly improved, increasing from 1.08 to 3.09%. Sago concentration significantly affected solvent retention, swelling, sedimentation properties, pasting temperature, and both peak and breakdown viscosities. At 50% fat replacement, the muffins exhibited increased density and firmness, measuring 32.67 N, compared to full-fat and lower-fat samples. Sensory evaluations by semi-trained assessors rated both full-fat and reduced-fat muffins within the liking range, with scores ranging from 8.52 to 7.42. Lightness values showed no significant difference between full-fat and reduced-fat muffins. These findings suggest that sago flour is an effective partial fat replacer in muffin formulations, enhancing nutritional value while maintaining acceptable sensory qualities, with the 25 and 37% replacements achieving the best balance of properties.

**Graphical abstract:**

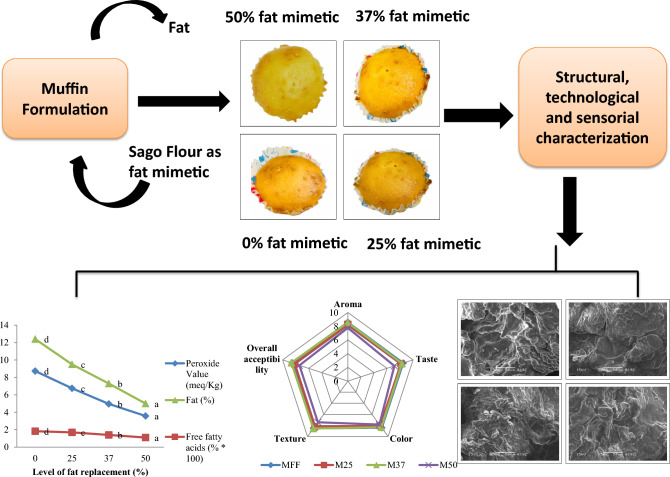

**Supplementary Information:**

The online version contains supplementary material available at 10.1007/s13197-024-06045-6.

## Introduction

Recently, researchers have been exploring innovative approaches to meet the increasing consumer demand of healthier food products and one of the approaches they have used is to reduce the fat content by employing fat mimetic (Moin et al. 2024).Fat is a macronutrient that plays a vital role in food matrices apart from basic nutrition. A wide variety of foods majorly depend on fat for improved palatability in order to meet consumer acceptance (Peng and Yao 2017). An array of fat formulations is used in the development of baked goods such as margarine, butter and shortening. Nonetheless, frequent consumption of bakery products contributes to the excessive intake of fat, which is a major contributor to excessive calorie intake and obesity (Hooper et al. 2015). Increased consumer concerns about full-fatbaked products such as cakes, muffins or biscuits have rushed the researchers and manufacturers to produce healthier and non-conventional alternatives of conventional snacks (Giuffrè et al. 2022). However, the other concerns regarding the elimination of fat may adversely influence the overall quality of product (Peng and Yao 2017).

A muffin is a kind of sweet and calorie dense baked product that is widely consumed because of its appealing taste and soft texture. Muffin batters are complex fat-in-water emulsion made up of a continuous phase of egg, sugar, water, and fat with a discontinuous phase of air in which flour particles are dispersed. A muffin comprises approximately 50% fat, which is responsible for its smooth and spongy texture (Martínez-Cervera et al. 2011).

A variety of fat replacers have been proposed to decrease the added fat content in food products while maintaining the sensory qualities associated with fat. Carbohydrate-based fat replacers are known as fat mimetic, basically obtained from cereals and other plants as these comprises both digestible and indigestible complex carbohydrate. Sago flour is obtained from sago palm (*Metroxylon sago*) which is traditionally utilized as a thickening and stabilizing agent in food industries (Md Zaidul et al. 2003). Sago is a nutritious, low caloric and easy to digest food, which is not traditionally used as fat replacer but its high nutritional properties, low calories and visosity make it worth to utilize as a carbohydrate based fat replacer.

This study explores the potential of sago flour as a viable alternative to fat in muffins, aiming to mitigate challenges linked to excessive fat intake from traditional full-fat formulations. By substituting butter with sago flour at different ratios, the research examines its impact on various quality characteristics of muffins, encompassing physical, physicochemical, textural and sensorial parameters. These findings contribute to advancing knowledge on healthier and more sustainable food options, potentially diversifying choices for future consumption patterns.

## Materials and methods

The raw materials utilized in the preparation of muffins including all-purpose wheat flour, commercial sago flour, salted butter, icing sugar, eggs, fresh buffalo milk yogurt and iodized salt were bought from a local supermarket in Karachi, Sindh, Pakistan. Analytical grade chemicals and solvents (Sigma-Aldrich) were utilized for analysis.

### Functional properties of flour

#### Swelling capacity

The swelling capacity of formulated flour was assessed by method outlined by Chandra et al. (2015).The flour sample was placed in a 100 mL graduated cylinder up to the 10 mL mark. Distilled water was then added to bring the total volume to 50 mL. The cylinder was inverted with its opening covered by a small petri plate to ensure thorough mixing. After standing for 2 min, the suspension was inverted again and allowed to stand for an additional 8 min. Finally, the increase in the sample’s volume was measured after the 8-min period.

#### Zeleny sedimentation test

The Zeleny sedimentation test was conducted in accordance with the guidelines provided in Method 56–60, approved by AACC (2000). In a 100 mL glass cylinder, 4 g of flour sample was moistened with 50 mL of 4 mg/L bromophenol blue solution. After being rapidly shaken for 5 sec, the sample was stirred for 5 min. Following that, a 25 mL isopropyl alcohol–lactic acid reagent was added, and the sample was again mixed in an agitator for 5 min. To make 1 L of the isopropyl alcohol–lactic acid reagent, 200 mL of isopropyl alcohol, 180 mL of lactic acid solution (25% v/v), and water were combined. The resulting suspension's volume was calculated in mL after it was allowed to stand for precisely 5 min. By multiplying the volume by the appropriate factor (0.95), the sedimentation value was obtained, allowing results to be expressed on a moisture basis of 14%.

$$\text{SDS}=\text{Sediment value }\times \text{flour moisture factor}$$

#### Solvent retention capacity profile

The SRC tests were carried out utilizing distilled water, 5% lactic acid, 5% sodium carbonate, and 50% sucrose in accordance with the procedure of Xiao et al. (2006).

#### Pasting properties of flour blends

The pasting properties of the flour blends with the addition of sago flour as fat replacer at 25, 37 and 50% were tested using a Rapid visco amylograph (Brabender, Duisburg, Germany). The method of Moin et al. (2016) was followed with slight modifications. The stainless-steel cup of amyloghraph was filled with a 15 g sample of flour, and 100 mL of water was added to make the flour blend suspension. Once the mixture had formed a slurry, it was stirred and attached to a Micro Visco Amylograph. The slurry was then stirred for 10 sec at 160 rpm and 50 °C. The slurry was heated for 7.3 min at 95 °C, and then it was heated for an additional 15.7 min at 95 °C, giving it a 5 min holding period. The slurry was cooled for 7.7 min after the temperature was lowered to 50 °C. Gelatinization temperature (°C), maximum viscosity (BU), setback (BU), hot paste viscosity (BU), cold paste viscosity and time to attain maximum (min) viscosity was recorded.

### Preparation of muffins

The muffins were prepared according to the method described by Martínez-Cervera et al. (2011). Four different muffin samples were baked to investigate the effect of varying fat replacement with sago flour. The M_FF_ sample served as the control, containing the maximum amount of fat (100%) and no sago flour. The remaining three samples, designated as M_25_, M_37_, and M_50_, had 25, 37, and 50% of their fat replaced with sago flour, respectively. Consequently, the flours used in the formulations of M_25_, M_37_, and M_50_ were composite flours of wheat and sago, containing 25 (WSF_25_), 37 (WSF_37_), and 50% (WSF_50_) sago flour, respectively, calculated based on the amount of fat replaced. The formulation ratios of muffins are depicted in Table S1. The conventional Dawlance Electric Oven (DWMO-4215 CR) was preheated at 175 °C for 30 min. Butter and sugar were beaten using a stand mixer of Kenwood KM280 for 3–4 min at top speed until a creamy texture was achieved. A small proportion of egg yolk, half milk and egg white were added and mixed it at low speed for 1 min and then poured the remaining portion of egg white and yolk and whisked it again for 3 min at maximum speed. The remaining dry ingredients (sago flour, sodium bicarbonate, salt and citric acid) were added and the batter was beaten for an additional minute at medium speed. Lastly the rest of the milk and fresh yogurt (for softer texture) were added and the batter was beaten for 3 min at medium speed until smooth. Prepared batter was poured into a paper moulds having 60 mm diameter and 36 mm height and arranged in a baking tray such a way that each row of tray contained each concentration and then baked for 20 min at 175 °C in an electric oven. After baking, the muffins were left to cool at room temperature for 1 h to prevent moisture condensing on the upper surface.

### Proximate analysis of muffins

#### Moisture content

The moisture content of muffins was determined using a standard method. A 10 g sample of the muffin was dried in a hot air oven (Model: DO-1-30/02, PCSIR, Pakistan) at a temperature of 105 ± 5 °C until a constant weight was obtained, following the AACC (2000) method No. 44-15.

#### Fat content

The fat content of the muffins was determined using the AACC (2000) Method No. 30-25. A Soxhlet extraction apparatus (Model: H-2 1045 Extraction Unit, Hoganas, Sweden) was employed for the assessment of crude fat content in both full-fat and reduced-fat muffins. Drided and crushed muffin sample (5 g) was placed in a thimble. For extraction 250 mL of n-hexane was used and heated at 65 °C on mantle heater. After 6 h, the solvent was evaporated by using rotary evaporator and the isolated fat was determined by the following formula:

$$\% {\text{Crude fat}} = \frac{{{\text{Weight of flask with extracted fat }}\left( g \right) - {\text{Weight of empty flask }}\left( g \right) }}{{{\text{Weight of muffin }}\left( g \right)}} \times 100$$

#### Protein content

The protein content in muffins was determined using the AACC (2000) method No. 46-30. Approximately 5 g of the muffin sample were digested in a Kjeltech device (Model: D-40599, Behr Labor Technik, GmbH-Germany) using 30% concentrated sulfuric acid and a catalyst. After digestion, the liberated ammonia was distilled into excess standard acid and titrated with a standardized base solution. The nitrogen percentage in the sample was determined by titrating the distillate against a 0.1 N H_2_SO_4_ solution until the color turned light golden. The crude protein content was calculated by multiplying the nitrogen percentage (N %) by the factor 6.25.

$$N\left(\%\right)=\frac{\text{Vol}.\text{ of }0.1\text{N H}2\text{SO}4\text{ x }0.0014\text{ x Vol}.\text{ of dilution }(250\text{mL})}{\text{Vol}.\text{ of distillate taken x Weight of sample}}\times 100$$

$$\text{Crude protein}\left(\%\right)=N \left(\%\right)\times 6.25$$

#### Ash content

The ash content of the muffin was determined by igniting 3 g powdered sample (db) in a Box type resistance muffle furnace (SX, China) at 550 °C for 6 h. Ash content was the remaining inorganic residue in the sample.

$$\text{Ash }(\%)=\frac{\text{Weight of inorganic residues}}{\text{Weight of sample}}\times 100$$

#### Crude fiber

The crude fiber content of muffins was analyzed by Gul and Safdar (2009). A sample weighing approximately 1.25 g was used. After adding 250 mL of a 1.25% sulfuric acid solution, the liquid was brought to a boil for 30 min. After the sample was filtered, the filtrate was heated for 30 min in 250 mL of 1.25% sodium hydroxide solution. The material was filtered again. After that, the residues were stored for an hour at 150 °C in a drying oven. After being allowed to cool in a desiccator, the residues were held at 550 °C for 3–4 h in a muffle furnace. After cooling the sample in desiccator, weigh it again. The following expression was used to determine the proportion of crude fiber in muffins:

$${\text{Crude}}\;{\text{fiber}}{\mkern 1mu} (\% ) = \frac{{{\text{Crucible}}\;{\text{weight with}}\;{\text{fiber }}\left( g \right) - {\text{Weight of}}\;{\text{crucible}}\;{\text{with ash }}\left( g \right)}}{{{\text{Sample weight }}\left( g \right)}} \times 100$$

#### Peroxide value

Peroxide value (PV) of muffins was determined by AOAC (2003) protocol.

#### Free fatty acids

The free fatty acids (FFA) fat samples extracted from muffins was determined by following AOAC (1995) methodology 969.33.

### Structural analysis of muffins by Scanning electron microscope (SEM)

Using the SEM technique, the microstructure of the muffins was assessed. With a few minor adjustments, Bhatt et al. (2021) method for sample preparation was followed. Muffins were first thinly sliced and then freeze-dried for five hours. The dried sample was then placed in polyethylene bags, sealed, and stored in a desiccator until needed again. The instrument used was a JEOL scanning electron microscope (Model JSM-6380 A) located at the University of Karachi's centralized science laboratories. Samples that had been freeze-dried were put on a specimen holder and sputter-coated with gold up to 300 A. Samples were then observed at a vacuum of 9.75 × 10^−5^ torr at 15 kV.

### Dimensional analysis and baking weight loss of muffins

The height of muffins was measured using Vernier calliper. The bulk density of muffin was measured as the ratio between weight of sample and its volume (AACC 2000) and the specific volume of muffin was measured by rapeseed displacement method (44-15.02) described by AACC (2000). The weight loss was calculated as the percentage difference between initial and final weight of muffin.

### Colour analysis

The colour of full-fat and reduced fat muffins was analyzed in terms of L*, a* and b* values by following the protocol of Ahsan et al. (2023).

### Texture analysis of muffins

The firmness of freshly prepared muffins was evaluated after cooling them to room temperature. Texture Profile Analyzer (Model: TA-XT. plus, Stable Micro Systems, Godalming, UK) was used. The test was performed using a cylindrical 36 mm-diameter aluminum probe and a 50 kg load cell. The highest force (N) required to compress the muffins by 50% depth at a test speed of 1 mm/sec was noted.

### Sensory evaluation

A panel of semi-trained assessors (*n* = 26) evaluated the muffins' sensory response using the 9-point hedonic scale system. The panelists were non-smokers and healthy adults, representing a 20–30 age group. Purpose of study, composition of samples, sensory attributes and necessary practices for sensory analysis were explained to them. They were allowed to leave the sensory evaluation even after participating in the short training. With the informed consent, panelists assessed the sensory qualities of muffins in terms of aroma, taste, colour, texture and overall acceptability. The score of 1 represented “extremely dislike” while a score of 9 represents “extremely like”. All samples were coded with three-digit random numbers and were served in a calm and bright environment. Moreover, clean drinking water was served with muffins to clean the oral cavity before tasting and scoring the next sample.

### Statistical evaluation

Statistical software (Statistix-8.1) was used to conduct analysis of variance (ANOVA) in order to determine the significance level (*p *> 0.05). Additionally, a post-hoc mean comparison was carried out using Tukey's honest significant difference.

## Results and discussion

### Physicochemical properties of flour composite

The physicochemical properties of wheat and wheat-sago composite flour were studied in terms of capacity of swelling (CSW), sedimentation (CS) and solvent retention (CSR) (Table 1). Statistically significant increment in CSW and CS was observed with the increase in addition level of sago flour. The swelling capacity for wheat flour was found 12.23 mL while CSW of wheat-sago composite ranged between 13.30 and 16.30 mL. Interestingly, 78, 108 and 136% increment was observed in the sedimentation value of wheat-sago composite flours when compared to wheat flour. The principal constituent of sago flour is starch, and its properties differ from the amalgamation of starch and gluten present in wheat flour. This unique nature of sago starch, including granule size and amylose-amylopectin ratio, influences its sedimentation behavior in liquid media (Tester et al. 2004). Hydration properties, such as water absorption and retention capacities, are integral to understanding the sedimentation behavior of sago flour (Gunaratne and Hoover 2002). The ability of sago flour to interact with water influences its behavior in liquid suspensions.

**Table 1:** Physicochemical properties of wheat and sago flour composites^a^

Samples	Swelling Capacity (mL)	Sedimentation (mL/g)	Lactic acid (%)	Solvent retention capacity		
				Sodium carbonate (%)	Sucrose (%)	Water (%)
WF	12.23 ± 0.31^d^	14.86 ± 0.65^d^	75.13 ± 1.41^c^	81.90 ± 1.57^c^	104.17 ± 2.41^d^	65.17 ± 1.25^c^
WSF_25%_	13.33 ± 0.57^c^	26.53 ± 0.62^c^	101.37 ± 2.06^b^	108.50 ± 2.75^b^	123.80 ± 2.65^c^	78.73 ± 1.72^b^
WSF_37%_	14.77 ± 0.74^b^	30.90 ± 0.46^b^	137.10 ± 1.35^ab^	134.40 ± 1.71^a^	133.60 ± 1.91^b^	113.07 ± 2.41^ab^
WSF_50%_	16.3 ± 0.42^a^	35.10 ± 0.33^a^	139.97 ± 2.37^a^	134.17 ± 0.85^a^	155.73 ± 1.79^a^	117.17 ± 0.93^a^

^a^WF: Wheat flour; WSF_25%_: 75% wheat and 25% sago flour; WSF_37%_: 63% wheat and 37% sago flour; WSF_50%_: 50% wheat and 50% sago flour. Mean ± standard deviation (SD) within the same column having different lowercase superscripts are significantly different

The solvent retention of flours was studied by using distilled water, lactic acid, sodium carbonate and sucrose solution. It is the amount of these solvents held by a flour sample after centrifugation and it depicts the swelling behavior of component polymers in the four solvents (Wessels et al. 2020). In general, the lactic acid retention in flour is associated with gluten strength, sodium carbonate retention with the presence of damaged starch, while sucrose retention is linked with pentosan characteristics. However, the water retention is subjective to all of these flour constituents. A significant (*p *≤ 0.05) sago concentration dependent increase was observed in the percent retention of all aforementioned solvents for wheat-sago composites when compared to all-purpose wheat flour. This could be due to difference in processing and composition of sago flour along with distinct molecular and structural properties of sago starch granule. The observed trend suggested that the proportions of damaged grain structures of starch and non-starch components was higher in sago flour. Flours with sedimentation values < 30 mL, between 30 and 60 mL, and higher than 60 mL are appropriate for making cookies, chapatti/pasta, and bread, respectively (Patil et al. 2015). In the present study, the WF and WSF_25%_ exhibited sedimentation value < 30 mL while WSF_37%_ and WSF_50%_ were found to have sedimentation value between 30 and 60 mL suggesting the application of these flour composites in biscuits, flat breads, pasta products and their variants.

### Viscosity profile of flour composite

The pasting properties of wheat and wheat-sago composite are summarized in Table 2. The term pasting refers to temperature-dependent variations in the viscosity of a hydrated system. The pasting temperature of wheat-sago composite ranges between 61.1 and 70.1 °C and it raised with the increase in amount of sago flour. The high pasting temperature point towards an elevated resistance of starch granular swelling and rupturing. Maximum enhancement in viscosity during heating, when the granules of starch are gelatinized completely is termed as peak viscosity (V_max_). It is achieved when the rate of granular swelling is equivalent to the granular breakdown. The V_max_ also increased with the increase in amount of sago flour. This is in accordance with sago concentration dependent increase swelling capacity wheat-sago composites (Table1). The findings are also consistent with the study of Md Zaidul et al. (2003) describing wheat flours having different protein content upon addition of sago exhibit sago concentration dependent V_max_. Moreover, increment in pasting temperature and V_max_ was also observed for wheat-sorghum blend when compared to wheat flour (Im et al. 1998). The effectiveness of polysaccharide-based fat replacers resides in their ability to modulate key functional properties, including viscosity enhancement, gel formation and amplified water-holding capacity (Bourouis et al. 2023). The breakdown and hot paste viscosities of wheat flour was found significantly higher (*p *≤ 0.05) than all three composite wheat-sago flours. The setback viscosity also increased with the level of sago flour addition. The lower setback viscosity principally implies lower degree of retrogradation (Moin et al. 2017).

**Table 2:** Pasting properties of wheat and sago flour composites^a^

Samples	PT (BU)	Wmax (BU)	Breakdown (BU)	HPV (BU)	CPV (BU)	Setback (BU)
WF	58.2 ± 2.67^c^	1011 ± 7.38^c^	418 ± 3.72^a^	888 ± 6.93^a^	1345 ± 9.34^d^	530 ± 2.39^c^
WSF_25%_	61.1 ± 3.98^b^	1066 ± 5.24^bc^	288 ± 2.59^b^	774 ± 4.28^c^	1463 ± 3.56^c^	536 ± 6.48^bc^
WSF_37%_	69.1 ± 2.15^a^	1075 ± 6.76^b^	271 ± 1.83^bc^	785 ± 5.81^bc^	1546 ± 5.72^b^	581 ± 2.17^b^
WSF_50%_	70.1 ± 4.54^a^	1081 ± 3.29^a^	258 ± 2.66^c^	796 ± 6.27^b^	1616 ± 6.39^a^	628 ± 5.38^a^

^a^WF: Wheat flour; WSF_25%_: 75% wheat and 25% sago flour; WSF_37%_: 63% wheat and 37% sago flour; WSF_50%_: 50% wheat and 50% sago flour. PT: peak temperature; Vmax: peak viscosity; HPV: hot paste viscosity and CPV: cold paste viscosity. Mean ± SD within the same column having different lowercase superscripts are significantly different

### Compositional analysis of muffins

The composition of full fat and reduced fat muffins is summarized in Table 3. Moisture content of muffins increased with the increased fat mimetic level which could be due to increased water retention capacity of wheat-sago composite flours (Table 1). Increased percent moisture is also reported for muffins when fat was replaced with soluble cocoa fiber (Martínez-Cervera et al. 2011) and commercial inulin (Zahn et al. 2010). The fat percentages were significantly reduced as the butter was replaced with sago flour whereas, mineral content increased after substitution of fat with sago flour. However, the increment in ash content was insignificant at lower level of fat substitution with sago flour (M_25_) while for M_37_ and M_50_ the significant improvement (*p *≤ 0.05) in amount of minerals were observed. Enhanced mineral content was also observed in muffins when fat was replaced with peach dietary fiber (Grigelmo-Miguel et al. 2001). Moreover, the percentage of crude fiber content in reduced-fat and full-fat muffins ranged between 0.49 and 0.50%, and the differences were insignificant among all muffins. The pH of muffins ranged between 5.15 and 5.90. Insignificant change in pH was observed among M_25_, M_37_ and M_50_ (Table 3). Furthermore, significant decline was observed in free fatty acid and peroxide values of muffins after butter replacement with sago and the trend was found as M_50 _< M_37 _< M_25 _< M_FF,_attributed to decline in added butter amount. This also highlights enhanced health and quality prospects of reduced fat muffins.

**Table 3:** Compositional analysis of full fat and reduced fat muffins^a^

Samples	Moisture (%)	Fat (%)	Ash (%)	Crude fiber (%)	Proteins (%)	pH	Peroxide value (meq/kg)	Free fatty acids (%)
M_FF_	23.60 ± 0.82^c^	12.40 ± 0.46^a^	1.08 ± 0.10^c^	0.50 ± 0.03^a^	3.08 ± 0.20^a^	5.51 ± 0.56^b^	8.73 ± 0.23^a^	0.03 ± 0.003^a^
M_25_	25.36 ± 1.24^bc^	9.50 ± 1.15^b^	1.30 ± 0.34^c^	0.52 ± 0.01^a^	2.81 ± 0.60^ab^	5.68 ± 1.01^ab^	6.75 ± 0.14^b^	0.02 ± 0.004^ab^
M_37_	29.80 ± 0.36^b^	7.30 ± 0.53^bc^	2.30 ± 0.46^b^	0.49 ± 0.09^a^	2.66 ± 0.57^c^	5.90 ± 0.70^a^	4.96 ± 0.28^c^	0.01 ± 0.003^b^
M_50_	32.60 ± 1.18^a^	5.17 ± 0.75^c^	3.90 ± 0.50^a^	0.51 ± 0.29^a^	2.51 ± 0.11^d^	5.72 ± 0.82^a^	3.57 ± 0.14^c^	0.01 ± 0.002^b^

^a^M_FF_: Full-fat muffins; M_25_: 25% fat replaced muffins; M_37_: 37% fat replaced muffins; M_50_: 50% fat replaced muffins. Mean ± SD within the same column having different lowercase superscripts are significantly different

### Microstructure of muffins

**Fig. 1:**
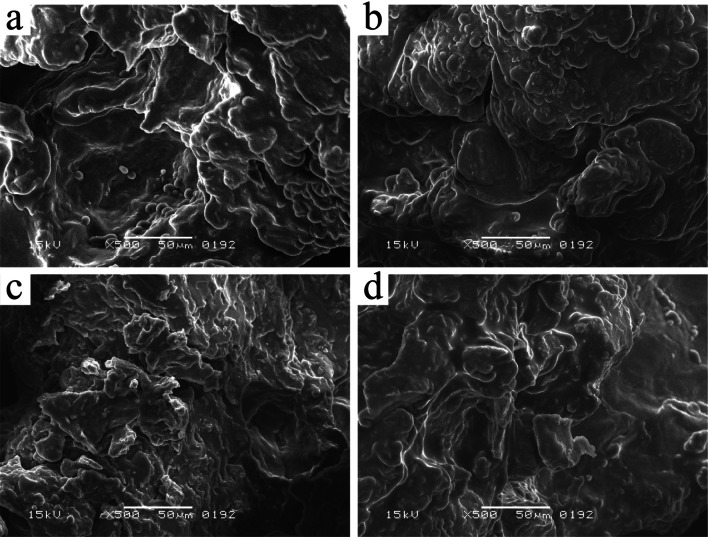
SEM images of **a** 25% fat replaced muffins; **b** 37% fat replaced muffins; **c** M_50_: 50% fat replaced muffins; **d** full-fat muffins at 500x

The SEM micrographs at 500x are represented in Fig. 1. The gelatinized starch granules embedded with protein matrix could be observed in M_25_, M_37_ and M_50_ however, in M_FF_ the starch granules are less visible and are probably covered with fat strands. Due the presences of fat in M_FF_ the structure appeared smoother than muffins containing sago flour. During baking, melted butter functioned as a surface lubricant, resulting in a smooth muffin appearance. However, increasing fat replacement levels led to an increasingly irregular matrix. Individual starch granules, instead of being embedded within the matrix, were observed as distinct structures on the surface. These structural variations are concordant with the observations of fat replacement by inulin in sponge cake (Rodríguez‐García et al. 2012). Fat present in protein–starch–fat networks lead to tender and softer texture to semi-solid or solid food systems (Peng and Yao 2017). Reduction in amount of fat lead to denser structure in muffins. This is also evident from bulk density and textural analysis findings (Table 4). Denser muffin structures are also attributed to fiber enrichment (Heo et al. 2019; Lee et al. 2015).

**Table 4:** Dimensional, textural and colorimetric properties of full fat and reduced fat muffins^a^

Sample	Height (cm)	Bulk density (g/cm^3^)	Specific volume (cm^3^/g)	Hardness (N)	Baking loss (g)	L*	a*	b*
M_FF_	30.79 ± 0.88^b^	0.59 ± 0.04^c^	3.89 ± 0.09^a^	29.21^ab^	15.20 ± 0.46^a^	63.67 ± 0.79^a^	− 1.51 ± 0.01^b^	− 1.53 ± 0.02^b^
M_25%_	31.49 ± 0.33^a^	0.67 ± 0.06^b^	3.26 ± 0.22^ab^	25.48^c^	12.25 ± 0.20^b^	63.56 ± 0.81^a^	− 1.58 ± 0.01^a^	− 1.65 ± 0.01^a^
M_37%_	31.85 ± 0.26^a^	0.71 ± 0.08^ab^	3.60 ± 0.22^ab^	27.16^b^	10.44 ± 0.51^bc^	63.49 ± 0.76^a^	− 1.58 ± 0.02^a^	− 1.67 ± 0.02^a^
M_50%_	30.16 ± 0.16^b^	0.77 ± 0.12^a^	2.96 ± 0.16^c^	32.67^a^	6.64 ± 0.35^c^	63.60 ± 0.37^a^	− 1.55 ± 0.01^ab^	− 1.62 ± 0.02^ab^

^a^M_FF_: Full-fat muffins; M_25_: 25% fat replaced muffins; M_37_: 37% fat replaced muffins; M_50_: 50% fat replaced muffins. Mean ± SD within the same column having different lowercase superscripts are significantly different. L* value depicts lightness (100 = white/0 = black), negative a* value depicts greenness, and (−) b* value highlights blueness

### Dimensional, textural and colorimetric analysis of muffins

The height (cm), bulk density (g/cm^3^) and specific volume (cm^3^/g**)** of muffins are among the most studied post baking parameters influencing both the consumer acceptability and quality of muffins, are summarized in Table 4. It was observed that replacement of fat had an insignificant influence on muffin height for M_50_ while upon lower fat replacement with sago (M_25_ and M_37_), smaller yet significant enhancement in height was observed when compared with M_FF_ sample. This shows that M_25_ and M_37_ had better baking expansion and gas retention compared to full fat and sago flour replaced with 50% of fat muffins. Furthermore, it is also evident with lower hardness of M_25_ and M_37_ than M_FF_ and M_50._ The bulk density was increased significantly after fat substitution with carbohydrate-based fat replacer (sago flour) and the trend was found as M_FF _> M_25 _≥ M_37 _> M_50_ attributed to increased post baking moisture retention upon fat substitution. Insignificant change in specific volume was observed for M_25_ and M_37_ when compared to M_FF_, however, it was significantly reduced for M_50_. A decrease in muffins volume was also observed when 50% baking fat was replaced by inulin (Zahn et al. 2010), 30–40% fat replaced with barley β-glucan concentrate and 20–40% baking fat replaced with oat β-glucan concentrate in cakes (Kalinga and Mishra 2009). The findings suggested fat replacement with sago flour over 37% will lead to formation of denser muffins with higher firmness (Table 4) attributed to the relatively stronger gluten network formation. During dough mixing, lipids function as lubricants and hinder gluten network formation. In the absence of shortening, the aqueous phase (water and sugar) readily interacts with flour proteins, leading to the development of a cohesive and extensible gluten network. However, when fat is present, lipid molecules physically coat the proteins and starch granules, impeding their interaction with water and consequently disrupting gluten network continuity (Mamat and Hill 2014). This interference of fat with gluten development is a key factor contributing to the textural characteristic of wheat-based bakery products.

Hardness is a primary textural attribute and it is described as force necessary to attain a given deformation. Firmness of prepared muffins ranged between 25 and 32 N. Increment in firmness of reduced fat muffins was observed with the increase in level of fat replacement. The denser texture is also evident with increased bulk density and lower specific volume of fat substituted muffins (Table 4). In the absence of lipids, protein networks within the dough exhibit enhanced development, marked by increased cross-links and a denser structure. Carbohydrate-based fat replacers could disrupt the formation of robust protein networks. However, due to their differing properties in terms of melting behavior (e.g. during baking) and recrystallization (e.g. upon coming to room or chilling), they may not fully replicate the functional role of lipids. This disparity can ultimately influence the textural attributes of the final product, potentially impacting its perceived crunchiness or creaminess (Peng and Yao 2017). Higher swelling capacity and water retention of the flour (WSF 25%) used in the preparation of M_25_ compared to wheat flour (Table 1) used in M_FF_ could lead to a softer texture in M_25_. Moreover, the presence of 75% fat in M_25_, when compared to higher fat substituted muffin samples, could hinder protein network development, consequently contributing to the softer texture of M_25_.

The baking losses significantly reduced (*p *≤ 0.05) with the level of fat replacement when compare to full fat muffins (Table 4). This could be due to higher water retention capacity of wheat-sago composites (Table 1).

Many cross-modal studies have linked the colour of edible commodities with their taste therefore, colorimetric attributes are considered as important quality parameters (Spence et al. 2015).The colour of full-fat and reduced fat muffins was studied in terms of L*, a* and b* values (Table 4). Statistically insignificant change was observed in L* and a* values of full-fat and reduced fat muffins. The b* values significantly reduced for reduced fat samples when compared to full fat muffins however, the change among different levels of fat substitution was statistically insignificant. On the contrary, significantly lower L* values (darker appearances) were observed when the baking fat was replaced with different β-glucan concentrates in cakes (Kalinga and Mishra 2009) and with peach dietary fibers in muffins (Grigelmo-Miguel et al. 2001).

### Sensory analysis of muffins

**Fig. 2:**
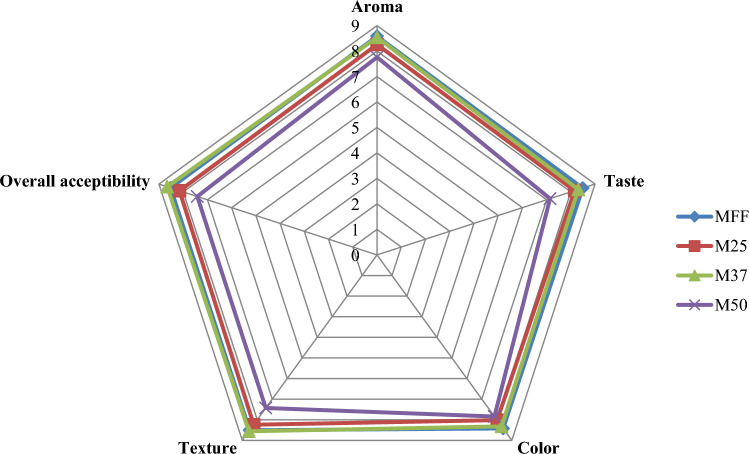
Hedonic scores of full-fat and reduced-fat muffins

The hedonic scores for aroma, taste, colour, texture and overall acceptability of both full-fat and reduced fat muffins samples were found in the liking or acceptability range (Fig. 2), suggesting the replacement of fat upto 50% with sago flour would not negatively affect the organoleptic characteristics of muffins. The development of sensory-acceptable reduced-fat foods is enabled by polysaccharides, which exhibit higher adhesion to sliding surfaces (tongue and palate) and a lower coefficient of friction (Bourouis et al. 2023). Furthermore, small yet significant reduction in hedonic scores were observed when fat was reduced to 50% when compared to M_FF_, M_25_ and M_37_ (*p *≤ 0.05). It could be due hard and denser texture (Table 4) and reduced buttery aroma of M_50_ due to 50% lower fat content in the formulation. Food odor have an impact on taste perception and food texture (Han et al. 2019).Textural attributes seem to exert a greater influence on consumer acceptance compared to flavor. Insignificant differences in all sensory parameters was observed between M_FF_, M_25_ and M_37_ suggesting up to 37% fat replacement with sago is acceptable by semi-trained panelists. Fat replacement with 100% carbohydrate based fat replacers (inulin or maltodextrin) exhibited significant negative impact on consumer acceptance of muffins (Zahn et al. 2010) and short dough biscuits (Błońska et al. 2014).

### Correlation of fat substitution with plant based fat replacer on compositional and quality characteristics of muffins

Pearson’s correlation of fifteen attributes of muffins was evaluated with the level of fat replacement by using sago flour as fat mimetic (Table S2). Strong positive correlation of increasing level of fat substitution was found with ash (0.848**), moisture (0.943**) and bulk density (0.700*) of muffins. The positive correlation with mineral content suggests sago based reduced fat muffin could help improve bone health and lower obesity risk. Minerals and vitamins have demonstrated potential in preventing bone diseases. High saturated fatty acids diet is associated with poor calcium absorption in animal studies probably due to the formation of non-digestible calcium and saturated fatty acids complexes in the intestine (Proia et al. 2021). A strong negative correlation (− 0.874**) was observed between fat and ash percentages for muffins, predicting enhanced health promoting attributes of butter replacement with sago. The negative strong correlation was observed for protein (− 0.757**), specific volume (− 0.764**), baking weight loss (− 0.967**), hardness (− 0.971**) and b* (− 0.691*) with fat substitution levels. This is also evident from the findings of ANOVA at (*p *≤ 0.05). The negative association with protein content was due to gluten protein dilution with starch rich sago flour while the improved water holding due to sago addition was the reason of lower baking weight loss in fat replaced muffins.

## Conclusion

The current study discovered the application of sago flour as a carbohydrate-based fat replacer in muffin preparations. The research revealed that textural and sensory features remained satisfactory with up to 37% replacement of butter with sago flour. These results recommend that sago flour holds aptitude in addressing increasing concerns linked to fat consumption while increasing the nutritional profile of bakery items. By utilizing inexpensive sago flour, this study supports the development of ready-to-eat low-fat bakery products such as cakes, pastries, and brownies. However, future studies can further discover optimization approaches and consumer acceptance to ease the commercialization of these products, contributing to broader health and culinary benefits.

## Supplementary Information

Below is the link to the electronic supplementary material.

Supplementary file1 (DOCX 17 kb)

## Data Availability

Data are new and are not previously published or submitted to other journals.

## Code availability

All data are incorporated in the tables.
