# Supplementary material for: Formulation and characterization of reduced fat muffins using a plant based fat replacer
Source: J Food Sci Technol. 2024 Sep 28;62(3):551–61. doi: 10.1007/s13197-024-06045-6 (PMC11794931; doi:10.1007/s13197-024-06045-6)
Supplement: Supplementary file 1 — Supplementary file1 (DOCX 17 kb) [file 13197_2024_6045_MOESM1_ESM.docx]

**Table S1 Composition of full-fat and reduced-fat muffins**

| **Ingredients** | **M_FF_** | **M_25_** | **M_37_** | **M_50_** |
| --- | --- | --- | --- | --- |
| Flour (g) | 100 | 100 | 100 | 100 |
| Sugar (g) | 100 | 100 | 100 | 100 |
| Yogurt (g) | 50 | 50 | 50 | 50 |
| Egg white (g) | 54 | 54 | 54 | 54 |
| Egg yolk (g) | 27 | 27 | 27 | 27 |
| milk (g) | 50 | 50 | 50 | 50 |
| Butter (g) | 46 | 34.5 | 29 | 23 |
| Sago flour (g) | ----- | 11.5 | 17 | 23 |
| Sodium bicarbonate (g) | 4 | 4 | 4 | 4 |
| Citric acid (g) | 3 | 3 | 3 | 3 |
| salt (g) | 1.5 | 1.5 | 1.5 | 1.5 |

Note: MFF: Full-fat muffins; M25: 25% fat replaced muffins; M37: 37% fat replaced muffins; M50: 50% fat replaced muffins

**Table S2 Pearson correlation of different levels of fat replacement with compositional and quality characteristics of muffins**

| **Parameters** | **Fat** | **Ash** | **Protein** | **Carbs** | **Fiber** | **Moisture** | **pH** | **Height** | **Bulk Density** | **Specific Volume** | **Baking Weight**  **Loss** | **Hardness** | **L*** | **a*** | **b*** |
| --- | --- | --- | --- | --- | --- | --- | --- | --- | --- | --- | --- | --- | --- | --- | --- |
| **Fat replacement level** | -.970^**^ | .848^**^ | -.757^**^ | .009 | -.696^*^ | .943^**^ | .181 | .374 | .700^*^ | -.764^**^ | -.967^**^ | -.971^**^ | -.066 | -.575 | -.691^*^ |
| **Fat** |  | -.874^**^ | .676^*^ | .014 | .674^*^ | -.939^**^ | -.227 | -.326 | -.712^**^ | .728^**^ | .961^**^ | .959^**^ | .069 | .470 | .628^*^ |
| **Ash** |  |  | -.631^*^ | .048 | -.877^**^ | .917^**^ | .068 | .077 | .714^**^ | -.680^*^ | -.920^**^ | -.923^**^ | -.104 | -.083 | -.308 |
| **Proteins** |  |  |  | -.454 | .643^*^ | -.754^**^ | -.143 | -.492 | -.339 | .401 | .773^**^ | .728^**^ | -.146 | .533 | .545 |
| **Carbohydrate** |  |  |  |  | -.161 | .058 | -.045 | -.055 | -.110 | .027 | -.022 | -.013 | .629^*^ | -.161 | -.130 |
| **Fiber** |  |  |  |  |  | -.872^**^ | .033 | .010 | -.538 | .447 | .769^**^ | .789^**^ | -.018 | -.006 | .127 |
| **Moisture** |  |  |  |  |  |  | .145 | .260 | .667^*^ | -.637^*^ | -.957^**^ | -.963^**^ | -.026 | -.364 | -.493 |
| **pH** |  |  |  |  |  |  |  | .058 | -.220 | .058 | -.144 | -.167 | .023 | -.241 | -.248 |
| **Height** |  |  |  |  |  |  |  |  | .172 | -.005 | -.286 | -.194 | -.403 | -.578^*^ | -.680^*^ |
| **Bulk density** |  |  |  |  |  |  |  |  |  | -.715^**^ | -.679^*^ | -.696^*^ | -.078 | -.193 | -.412 |
| **Specific volume** |  |  |  |  |  |  |  |  |  |  | .748^**^ | .789^**^ | -.030 | .368 | .459 |
| **Baking weight loss** |  |  |  |  |  |  |  |  |  |  |  | .989^**^ | .079 | .389 | .518 |
| **Hardness** |  |  |  |  |  |  |  |  |  |  |  |  | .029 | .395 | .512 |
| **L*** |  |  |  |  |  |  |  |  |  |  |  |  |  | -.163 | .126 |
| **a*** |  |  |  |  |  |  |  |  |  |  |  |  |  |  | .887^**^ |
